# Estimation of Compression Depth During CPR Using FMCW Radar with Deep Convolutional Neural Network

**DOI:** 10.3390/s25195947

**Published:** 2025-09-24

**Authors:** Insoo Choi, Stephen Gyung Won Lee, Hyoun-Joong Kong, Ki Jeong Hong, Youngwook Kim

**Affiliations:** 1 Department of Electronic Engineering, Sogang University, Seoul 04107, Republic of Korea; dlstn3462@sogang.ac.kr; 2 College of Medicine, Seoul National University, Seoul 03080, Republic of Korea; leestephengyungwon@snu.ac.kr (S.G.W.L.); ssberg@snu.ac.kr (K.J.H.)

**Keywords:** deep convolutional neural network (DCNN), cardiopulmonary resuscitation (CPR), Doppler frequency, frequency-modulated continuous-wave (FMCW) radar, micro-doppler signature, regression, Wigner–Ville distribution (WVD)

## Abstract

**Highlights:**

**What are the main findings?**

- A novel method using frequency-modulated continuous-wave radar enables remote measurement of chest compression depth during Cardiopulmonary Resuscitation (CPR);
- Deep convolutional neural network (DCNN) models trained on Wigner–Ville distribution spectrograms achieved the lowest RMSE of 0.447 cm, improving accuracy by 11.5% compared to short-time Fourier transform-based DCNNs.

**What is the implication of the main finding?**

- The proposed method can be integrated into consumer devices like smartphones for real-time CPR monitoring in out-of-hospital cardiac arrest scenarios;
- Accurate remote measurement of chest compression depth during Telecommunication-CPR can enhance CPR quality and improve patient survival rates.

**Abstract:**

Effective Cardiopulmonary Resuscitation (CPR) requires precise chest compression depth, but current out-of-hospital monitoring technologies face limitations. This study introduces a method using frequency-modulated continuous-wave (FMCW) radar to remotely and accurately monitor chest compressions. FMCW radar captures range, Doppler, and angular data, and we utilize micro-Doppler signatures for detailed motion analysis. By integrating Doppler shifts over time, chest displacement is estimated. We compare a regression model based on maximum Doppler frequency with deep convolutional neural networks (DCNNs) trained on spectrograms generated via short-time Fourier transform (STFT) and the Wigner–Ville distribution (WVD). The regression model achieved a root mean square error (RMSE) of 0.535 cm. The STFT-based DCNN improved accuracy with an RMSE of 0.505 cm, while the WVD-based DCNN achieved the best performance with an RMSE of 0.447 cm, representing an 11.5% improvement over the STFT-based DCNN. These findings highlight the potential of combining FMCW radar and deep learning to provide accurate, real-time chest compression depth measurement during CPR, supporting the development of advanced, non-contact monitoring systems for emergency medical response.

## 1. Introduction

Out-of-hospital cardiac arrest (OHCA) frequently occurs, and the timely administration of cardiopulmonary resuscitation (CPR) can increase the likelihood of patient survival. In such situations, telecommunication CPR (T-CPR) plays a crucial role, allowing emergency responders to provide real-time guidance on proper compression posture and location via video calls [1,2].

In real emergency scenarios, adjusting compression posture and location based on video guidance from emergency personnel is relatively straightforward. However, achieving and maintaining the correct compression depth—which is a key determinant of CPR quality—remains a major challenge. Numerous clinical studies and international guidelines emphasize that failure to reach the recommended depth results in reduced perfusion and poorer outcomes [3,4]. Therefore, to maximize the effectiveness of CPR, a technology that enables reliable real-time measurement of chest compression depth in out-of-hospital environments is essential [5,6,7].

Research on measuring chest compression depth during CPR has been conducted using various methods [8,9,10,11]. In ref. [8], two accelerometer sensors were used to measure compression depth by double-integrating the acceleration, achieving relatively low error. Additionally, studies utilizing impulse-radio ultra-wideband radar, which is widely used in the medical field, have proposed a method where a transmitter is placed on the chest and a receiver on the back, measuring time delay variations caused by chest compressions to estimate depth [10,11]. This method offers high resolution and achieves an average error of 0.12 cm, demonstrating high accuracy.

However, these methods have limitations in OHCA, where necessary equipment is not readily available. To address this issue, ref. [9] proposed a method that uses accelerometer sensors embedded in smartphones and smartwatches to measure chest compression depth in the same way as [8]. This approach showed that smartphones and smartwatches recorded low error rates of 0.5 cm and 0.2 cm, respectively, enabling relatively accurate measurements.

Nevertheless, the smartphone-based method requires placing the device between the hands during CPR, which may potentially affect compression accuracy. Similarly, the smartwatch-based approach may be impractical if the responder does not own a smartwatch. Furthermore, in real emergency scenarios, rescuers frequently switch roles to avoid fatigue. During such role-switching, requiring the new provider to reattach or reposition a device could introduce delays and data discontinuity. These factors may limit the practicality of current wearable-based methods for ensuring continuous, uninterrupted monitoring in actual OHCA situations.

In this paper, we propose using frequency-modulated continuous-wave (FMCW) radar for the remote estimation of the depth of the chest compression during CPR. FMCW radar has been widely utilized in diverse domains, owing to its advantages in short- to mid-range sensing. In medical contexts, FMCW radar has already been used to monitor heart rate, respiration, and human motion. While current smartphones do not employ radar for medical sensing, consumer devices such as the iPhone 11 already embed ultra-wideband (UWB) radar modules for spatial awareness and communication. Furthermore, recent research has increasingly investigated radar-based sensing under smartphone setups [12,13,14,15,16,17], supporting the feasibility of radar integration for health monitoring applications. Thus, the need for remote chest compression measurement, especially in OHCA scenarios, indicates the potential value of our approach.

In this study, we estimate chest compression depth by integrating the Doppler frequency obtained from micro-Doppler spectrograms. Additionally, we employ artificial intelligence techniques to estimate chest compression depth. A regression model using the maximum frequency of the micro-Doppler spectrogram from a single chest compression and a convolutional neural network (CNN) model utilizing spectrogram images were implemented. Furthermore, to enhance CNN performance, the Wigner–Ville distribution (WVD) was applied to improve the resolution of the spectrogram.

To verify the model’s performance, eight human participants performed chest compressions on a mannequin under a total of 36 different measurement conditions. The experiment was conducted by combining four compression depths (3 cm, 4 cm, 5 cm, and 6 cm), three compression rates (90, 100, and 110 Compressions Per Minute, CPM), and three radar positions (chest, side, and head). The accuracy of chest compression depth measurement using FMCW radar was validated by comparing it with reference data.

This study proposes a method for remotely compensating for inaccurate chest compressions caused by a lack of equipment during T-CPR in OHCA scenarios. To achieve this, we developed a radar-based remote chest compression depth measurement technique and validated its performance through various approaches.

## 2. Related Theories

### 2.1. FMCW Radar Signal Model

**Figure 1:**
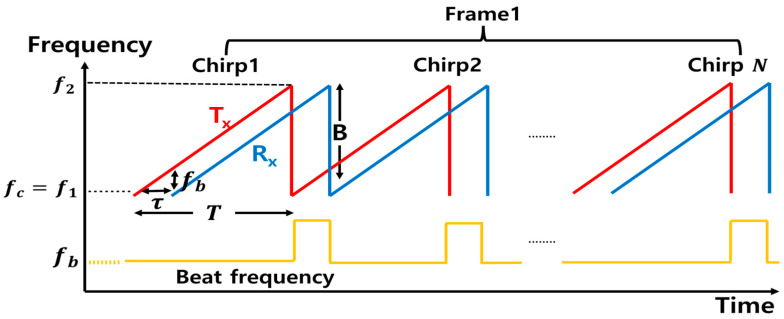
FMCW radar waveform.

FMCW radar uses a linearly changing frequency to measure a target’s information. FMCW radar determines range through beat frequencies $f_{b}$. The received signal is a delayed version of the transmitted signal, and the frequency difference, called the beat frequency, is proportional to the target’s range. The rising chirp signal linearly increases in frequency from $f_{1}$ to $f_{2}$ over chirp time T, as shown in Figure 1. The transmitted signal can be expressed as

(1) $$S_{T}\left(t\right)=A_{T}\cdot \cos[2\pi \left(f_{c}t+\frac{1}{2}S_{w}t^{2}\right)]\;S_{w}=\frac{B}{T}$$

$A_{T}$ is the amplitude; $f_{c}$ is the carrier frequency; $S_{w}$ is the frequency slope, and B is the bandwidth.

The received signal has the same structure as the transmitted signal but includes a time delay τ term, and it is expressed as

(2) $$S_{R}(t)=A_{R}\cdot S_{t}(t-\tau )\;\tau =\frac{2(R-vt)}{c}$$

$A_{R}$ is the received signal amplitude; R is the target distance; v is the target velocity, and c is the speed of light. The received signal $S_{R}(t)$ is mixed with the transmitted signal $S_{T}\left(t\right)$. This signal is then passed through a low-pass filter (LPF) to remove the high-frequency components, preserving only the difference signal $S_{IF}(t).$ The intermediate frequency (IF) signal for chirp 0 is expressed as

(3) $$S_{IF}(t)=\frac{A_{R}A_{P}}{2}\left\{cos\left[2\pi \left(S_{w}\tau t+f_{c}\tau -\frac{1}{2}S_{w}\tau^{2}\right)\right]\right\}$$

By performing the Fast Fourier Transform on $S_{IF}(t)$, we can obtain the value of $S_{w}\tau$. As the value of $S_{w}$ is known, the distance to the target can be calculated using the formula cτ/2 [18]. After the FFT process, a peak appears at the target’s location. By analyzing the phase information that changes across frames, the micro-Doppler effect can be identified and extracted. Here, a frame refers to one radar measurement cycle, which consists of multiple chirps transmitted and received sequentially.

**Figure 2:**
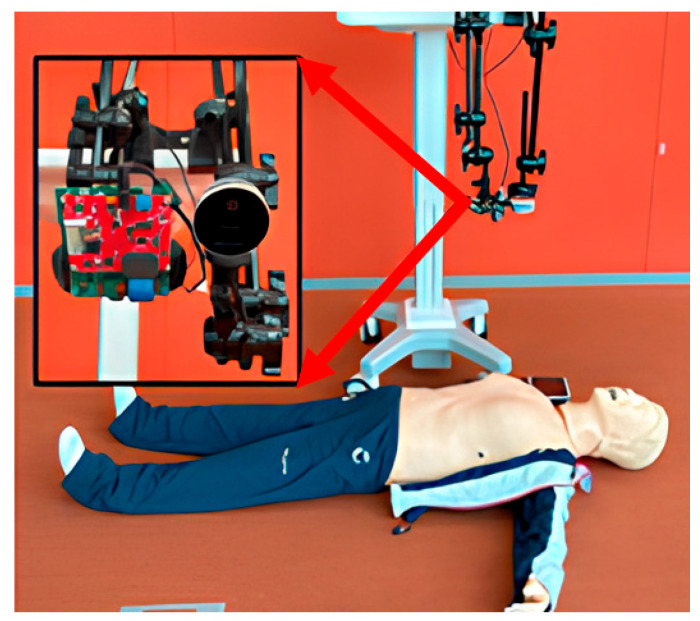
CPR measurement setup using a mannequin.

Using FMCW radar, we have measured chest compression for initial tests. The measurement setup is shown in Figure 2, and the radar parameters are summarized in Table 1. To ensure sufficient resolution for detecting chest displacements, we used a bandwidth of 3.99 GHz, which is the maximum supported by the radar hardware. While this provides improved range resolution, it may also increase susceptibility to noise, which was considered in the overall system design. Specifically, we employed the TI AWR1243, a 77-GHz FMCW radar with 12 dBm transmitting power developed by Texas Instruments.

### 2.2. Micro-Doppler Spectrogram

When a target undergoes small periodic movements, it generates micro-Doppler frequencies, which are fine-scale variations in Doppler shifts induced by localized or repetitive motion [19,20]. To investigate the Doppler signatures over time, we employ the Short-Time Fourier Transform (STFT) for joint time–frequency analysis. The STFT is a widely used linear time–frequency representation that analyzes non-stationary signals by applying a Fourier transform to short overlapping time windows. One of its key advantages is that it provides a cross-term free spectrogram, making it easier to interpret in practice. In addition, STFT offers computational simplicity, which makes it suitable for practical real-time applications [21,22,23,24]. The corresponding equation is shown below.

(4) $$STFT(t,\omega )=\int s(\tau )\cdot \gamma *(\tau -t)e^{-j\omega \tau }d\tau$$

which is an inner product and reflects the similarity between the signal s(τ) and the function of $\gamma (\tau -t)e^{jwt}$. The function γ is the window function that is only valid over a very short period, and thus, the inner product result has local frequency characteristics of the signal [21,23].

**Figure 3:**
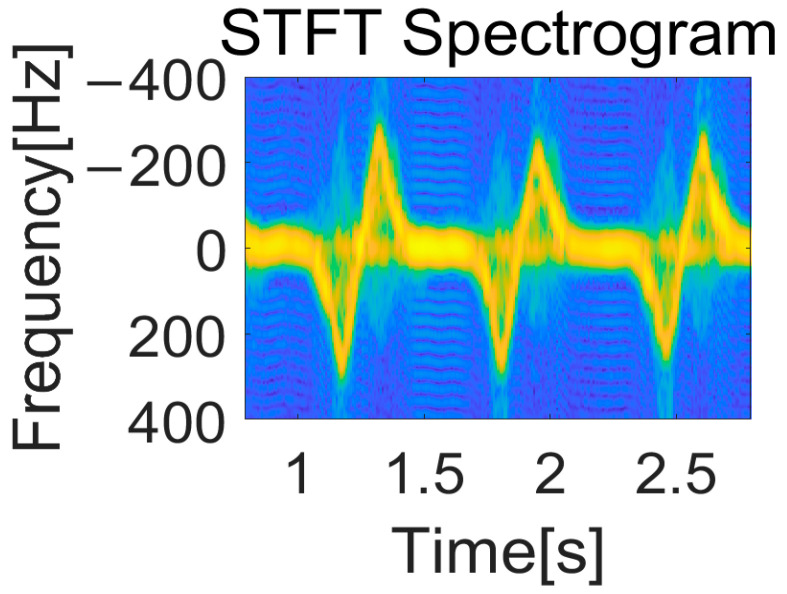
Spectrogram of CPR using STFT method.

An example of a spectrogram for CPR motion is demonstrated in Figure 3. We can observe the clear micro-Doppler signature of the compressed chest motion. The moment when the chest is compressed while moving away from the radar corresponds to the positive frequency region in the spectrogram. On the other hand, the moment when the compressed chest returns and moves closer to the radar corresponds to the negative frequency region in the spectrogram. In this context, the sign of the Doppler frequency directly reflects the velocity of the chest relative to the radar. A positive Doppler shift indicates motion away from the radar, and a negative Doppler shift indicates motion toward the radar. This bidirectional interpretation allows the spectrogram to capture both the downstroke and upstroke of each chest compression cycle.

**Figure 4:**
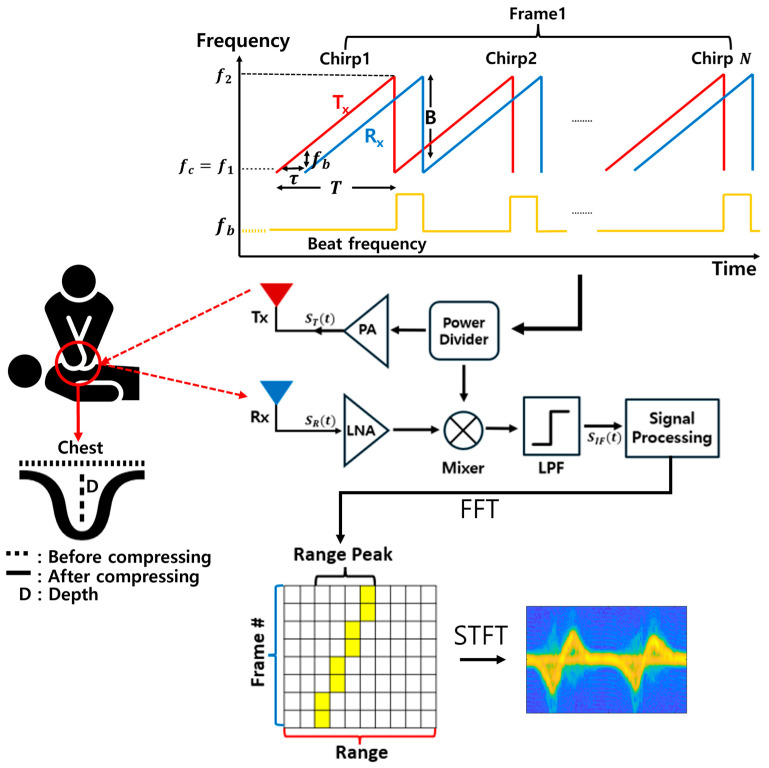
Overall process from chest compression to spectrogram generation.

Figure 4 illustrates the sequential process of generating spectrograms from chest compression signals. The received signal $S_{R}(t)$, captured by the receiving antenna Rx, is amplified by a low-noise amplifier (LNA) and then mixed with the reference signal in the mixer. This process generates an IF signal, which is subsequently passed through an LPF to remove high-frequency components. The resulting IF signal is used for range and velocity estimation after digital signal processing.

## 3. CPR Measurement Campaigns

**Figure 5:**
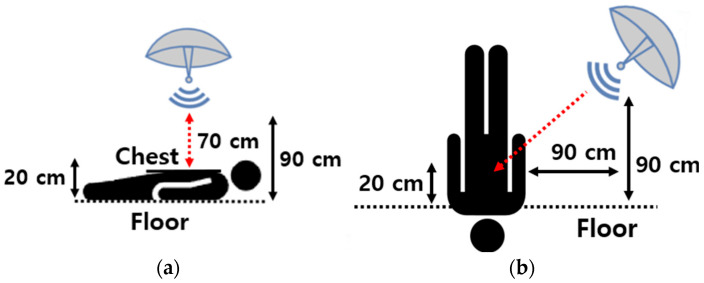

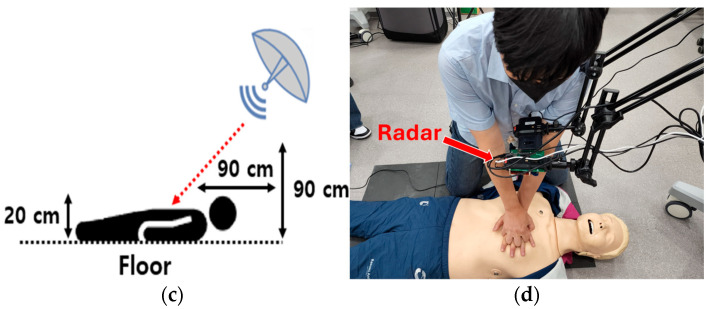
(**a**) Radar configuration of Case 1, (**b**) Radar configuration of Case 2, (**c**) Radar configuration of Case 3, and (**d**) Actual measurement setup.

Chest compressions were measured in three different cases according to the radar position. Case 1 was above the chest; case 2 was at the side of the chest, and case 3 was near the head. Photos depicting the radar position and distance for each experiment are presented in Figure 5.

In this measurement campaign, eight human participants performed chest compressions on the Resusci Anne QCPR mannequin (Laerdal, Stavanger, Norway). For each radar configuration, a total of 12 different measurement scenarios are conducted, targeting depths of 3 cm, 4 cm, 5 cm, and 6 cm, along with compression rates of 90, 100, and 110 per minute. Each scenario was measured for 30 s. To ensure compliance with the study protocol, participants were guided to maintain the specified chest compression depth and rate by using a SimPad (Laerdal, Stavanger, Norway), which displayed real-time compression depth measured by the mannequin. A metronome was used to help match the required compression rate. This study was approved by the Institutional Review Board (IRB no.2207-157-1344).

**Figure 6:**
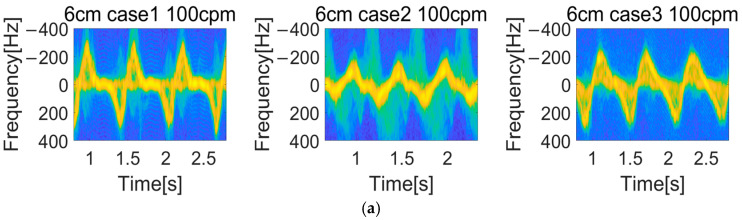

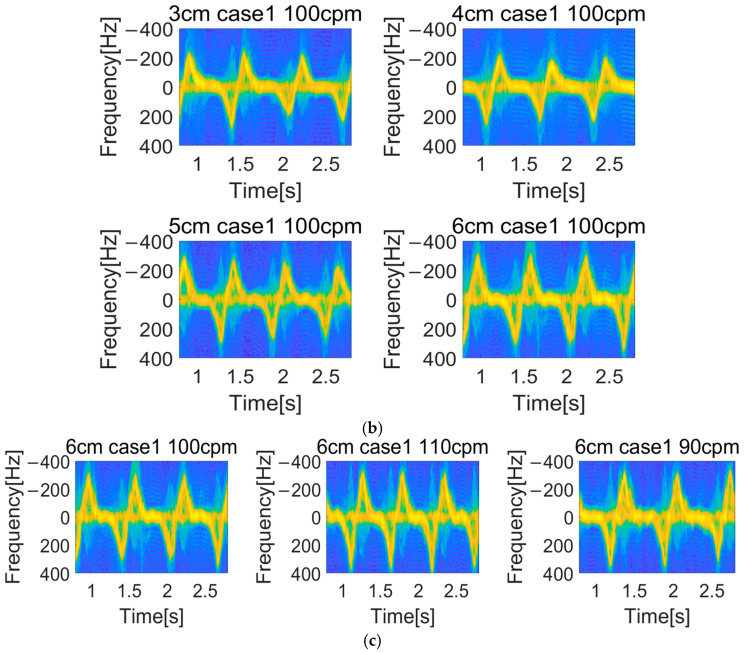
Spectrogram of different measurement campaigns: (**a**) Radar position variation (Cases 1–3), (**b**) Depth variation (3–6 cm), (**c**) Rate variation (90–110 CPM).

Figure 6a shows the spectrograms based on the radar position when the compression depth and rate are fixed. Compared to Case 1, it can be observed that Case 2 and Case 3 exhibit lower Doppler frequency components in the spectrogram, which indicates that chest motion appears relatively reduced due to the oblique radar angles. Figure 6b represents the spectrograms based on chest compression depth. In the given figure, the Doppler frequency varies with the compression depth. When pressing deeper for the same time duration, the speed of the hands for compression varies, leading to a higher maximum Doppler frequency in the spectrogram. Figure 6c illustrates the spectrograms based on compression rate. As the rate increases from 90 CPM to 110 CPM, it can be noted that there are more waveforms present for the same time frame.

**Figure 7:**
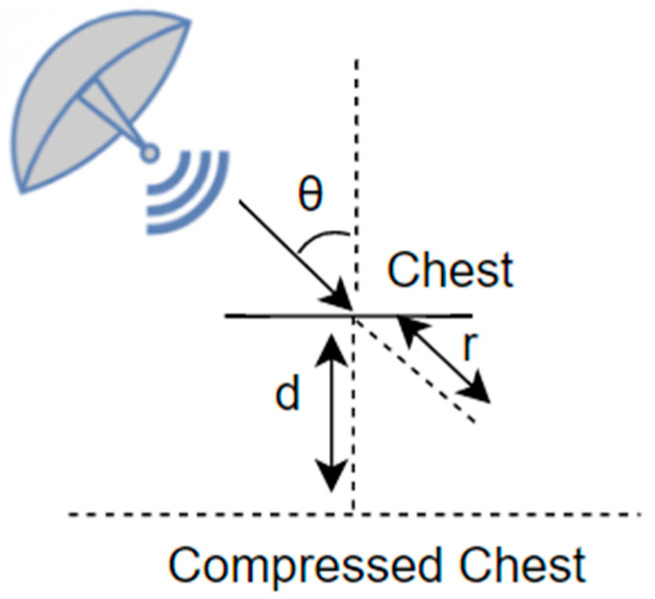
Aspect angle compensation.

It is important to note that when measuring a target positioned diagonally, as in Case 2 and Case 3, compensation must be applied because radar only measures radial displacement. In Figure 7, r represents the actual measured distance by radar; d represents the displacement through chest compression, and θ is the angle between the radial direction of radar and the normal direction of the chest in degrees. The compensation is performed by dividing the cosine of the measured displacement, and it can be expressed as

(5) $$\frac{r}{\cos\theta }=d$$

## 4. Depth Estimation Through Doppler Integration

Although the movement of range bins can be used to estimate the target’s distance, the displacement resolution is constrained by radar parameters, which in our setup corresponds to 3.75 cm. To overcome this limitation, we estimate displacement not from discrete range-bin shifts but from the continuous phase variation in the radar signal. These phase changes are reflected as micro-Doppler frequency components, which represent the instantaneous velocity of chest motion and correspond to the derivative of phase over time. Therefore, we employ the STFT spectrogram to extract the micro-Doppler frequency and integrate it over time to obtain the displacement caused by chest compressions. In other words, the phase information can be derived through Doppler integration, which not only accommodates range-bin movement but also acts as a smoothing process to reduce noise sensitivity and improve measurement accuracy [25]. The relationship between phase change and the target displacement is expressed as

(6) $$f=\frac{1}{2\pi }\cdot \frac{d\phi }{dt}\;\;\phi =k\cdot 2d=\frac{2\pi }{\lambda }\cdot 2d\;d=\frac{\lambda }{4\pi }\cdot \phi =\frac{\lambda }{2}\cdot \int fdt$$

where f is a frequency; ϕ is a phase; d is a displacement; λ is a wavelength, and k is a wave number.

**Figure 8:**
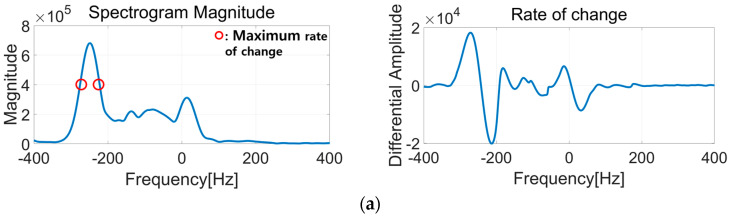

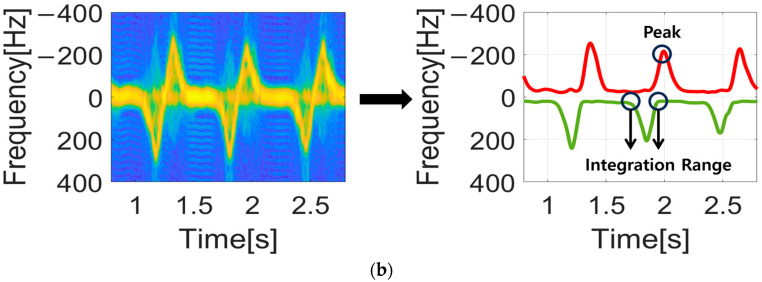
(**a**) Energy change with Doppler frequency at one time instance and (**b**) detected envelope from the spectrogram.

To integrate the Doppler signatures, it is necessary to identify the boundary of Doppler signals and separate them from noise in the spectrogram. However, the boundary between the Doppler signal and noise is blurry, which makes it difficult to determine the exact Doppler frequency. To address this issue, we proposed an envelope detection that finds the point with the highest differentiation value of energy over frequency, where the biggest energy change occurs, as shown in Figure 8a. The extracted envelope is presented in Figure 8b. After envelope detection, we defined the integration intervals based on the adjacent local minima of the waveform, as illustrated in Figure 8b, and used these intervals to calculate the displacement of chest compressions. At the same time, we also performed peak detection within each interval, and the extracted peak values were later used as input variables for the regression model described in Section 5.

**Figure 9:**
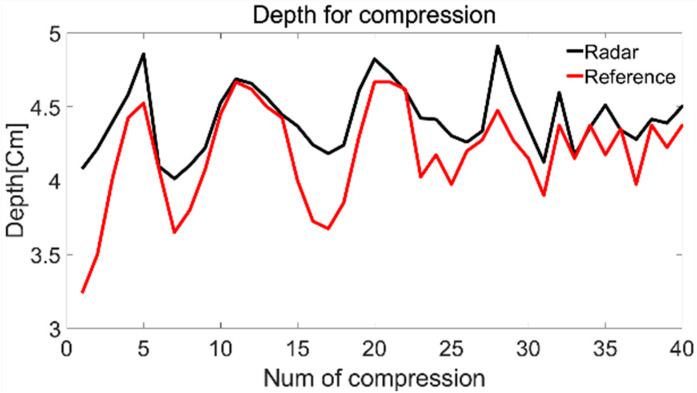
CPR compression depth through integration method.

Figure 9 shows the chest compression depth estimated per compression over 40 compressions using Doppler integration for a single subject at a rate of 110 CPM. The black line represents radar-estimated depth for each compression, while the red line indicates the corresponding reference values from the SimPad system. Although there are errors, it can be observed that the black line closely follows the shape of the red line.

We calculated the depth for each measurement and evaluated the performance. Average root mean square error (RMSE) values across subjects and cases are summarized in Table 2. All results are presented as averages over different CPMs.

The mean RMSE for Case 1 is 0.558 cm with a standard deviation of 0.179 cm, which is lower than that of Case 2 and Case 3. This indicates that, among the three radar positions tested, Case 1 tends to yield more accurate and consistent depth estimations. In addition, the confidence intervals show relatively narrower ranges for Case 1 in performance across different subjects compared to Cases 2 and 3.

The larger RMSE values in Case 2 and Case 3 suggest that non-frontal radar angles significantly degrade the accuracy of depth estimation. This also reflects the difficulty of compensating for real-time angular variations during Doppler integration. Therefore, additional strategies will be required to improve robustness against varying measurement angles in practical applications.

## 5. Depth Estimation Using Maximum Doppler Frequency Through Polynomial Regression

In this section, we suggest a method to estimate the compression depth using only the maximum Doppler frequency with a polynomial regression model. To achieve the correct CPR, higher compression speed and greater compression power are typically required, which leads to a higher Doppler frequency. As the compression depth increases, the maximum Doppler frequency also tends to rise. In addition, using the maximum Doppler frequency rather than the full waveform reduces the effect of noise and simplifies computation. Therefore, there is a high correlation between the highest Doppler frequency and the compression depth, which can be modeled by a polynomial regression model, which is a statistical technique that analyzes and models the relationship between input and output data through polynomials. The predicted outcomes of regression are typically continuous numerical values, and the performance of the model is evaluated based on the prediction error for a given set of data [26].

(7) $$E(w)=\frac{1}{N}\sum_{n=1}^{N}(w^{T}x_{n}-y_{n}{)}^{2}=\frac{1}{N}{\left\|Xw-y\right\|}^{2}\;\;\nabla E(w)=\frac{2}{N}X^{T}(Xw-y)=0\;X^{T}Xw=X^{T}y,w=(X^{T}X{)}^{-1}X^{T}y$$

Equation (7) represents a regression analysis, where w represents the weight; x represents the input variable, and y represents the actual value. The first line represents the mean squared error (MSE) cost function for linear regression, which we aim to minimize to find the best-fitting line for the data. To achieve this, we compute the gradient with respect to w in the second line. Setting this gradient to zero provides the conditions for minimum error, helping us find the optimal w that minimizes the cost function. From this step, the third line is derived, offering a closed-form solution for the optimal weight vector w in linear regression. By solving this equation, we obtain the best w that minimizes the errors for the given data.

**Figure 10:**
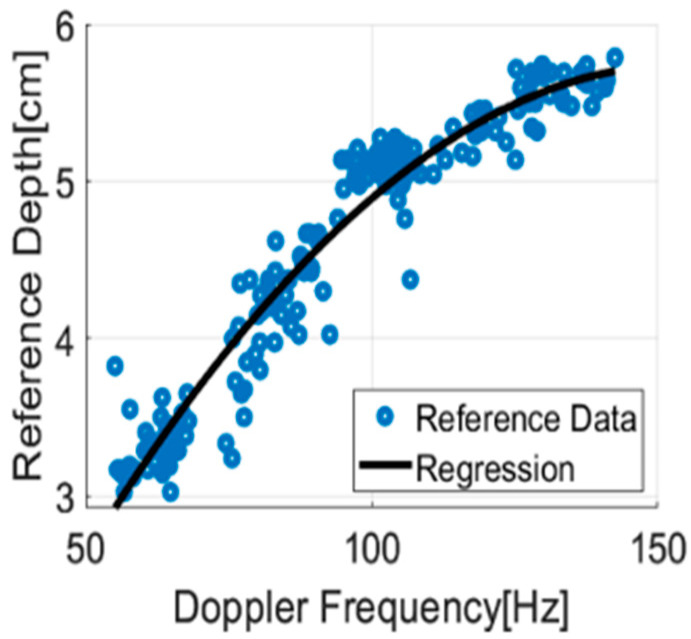
Regression model result.

In this study, we have utilized a second-order polynomial regression model to predict the compression depth based on the maximum Doppler frequency detected in the spectrogram. In Figure 10, the *x*-axis represents the maximum Doppler frequency, and the *y*-axis represents the corresponding compression depth (reference). The blue dots are reference data, and the black line is the regression model. The regression model is a simple method that can predict the depth of compression using the maximum Doppler frequency. The regression model is trained using data from 7 subjects, with the remaining 1 subject used for testing. This process is repeated for each subject, and the RMSE is calculated based on these models across all CPMs and cases. Table 3 summarizes the average RMSE (cm) for the regression model across subjects and radar positions.

Case 1 shows a mean RMSE of 0.506 cm, a standard deviation of 0.123 cm, and a 95% confidence interval from 0.403 cm to 0.609 cm. Case 2 shows a mean RMSE of 0.569 cm, a standard deviation of 0.169 cm, and a 95% confidence interval from 0.428 cm to 0.710 cm. Case 3 shows a mean RMSE of 0.533 cm, a standard deviation of 0.184 cm, and a 95% confidence interval from 0.379 cm to 0.687 cm. Between-subject variability is smallest in Case 1 and largest in Case 3, as indicated by the standard deviations and by the observed ranges across subjects of 0.365 to 0.753 cm in Case 1, 0.318 to 0.799 cm in Case 2, and 0.241 to 0.752 cm in Case 3.

Compared with the integration method, the regression model shows lower mean RMSE in Case 1, Case 2, and Case 3. The regression model also exhibits smaller standard deviations and narrower 95% confidence intervals.

## 6. Depth Estimation Using DCNN with STFT and WVD Spectrogram

### 6.1. DCNN to Spectrogram

This study proposes estimating chest compression depth using CNN. CNN is a type of deep learning model widely used in the fields of image processing, identification, and classification [27]. In radar fields, CNN models have been extensively applied for radar images, such as micro-Doppler signatures, Range-Doppler diagram, SAR, and ISAR.

CNN is composed of an input layer, several hidden layers including convolutional layers, activation functions, pooling layers, and an output layer. The strength of CNN lies in its ability to effectively learn local features, allowing it to recognize various objects or elements. Additionally, the learned filters possess translation invariance, enabling them to recognize the same features in different locations. In this study, the rectified linear unit (ReLU) was used as the activation function to enhance nonlinearity and improve training efficiency [28]. In addition, the extracted features can be used for regression purposes. DCNN refers to Deep CNN, emphasizing multiple hidden layers.

Now, the waveform in the spectrogram corresponding to a single chest compression is extracted from each spectrogram. To ensure a fair and unbiased evaluation of our CNN model, we carefully designed our dataset split to prevent any overlap between training, validation, and test sets. In this process, the model was trained separately based on different CPM and radar positions. A total of seven subject data sets were used for training and validation, while the data from one subject was completely excluded from both and used solely as the test set to evaluate the model’s generalization performance. The training and validation sets were further divided to monitor the learning process, and validation loss was used to apply early stopping to prevent overfitting. The model was trained using the Stochastic Gradient Descent with Momentum optimization algorithm [29], and the final model weights were selected from the epoch that yielded the lowest validation loss.

**Figure 11:**
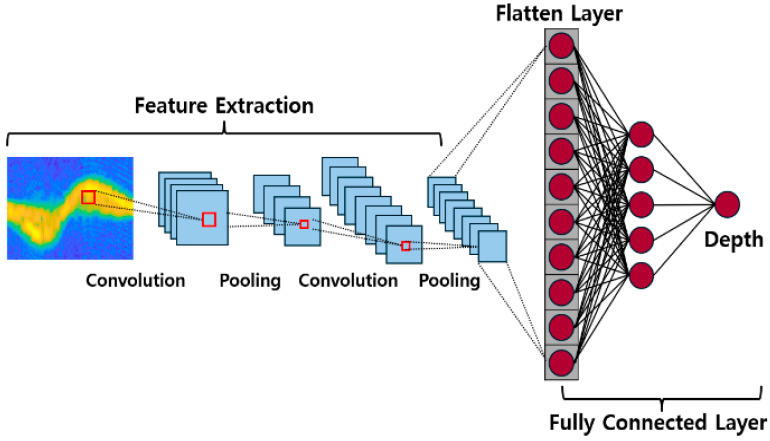
The compression depth estimation through DCNN.

To improve the accuracy of DCNN models, hyperparameters such as the number of layers and neurons per layer, as well as learning parameters, including learning rate and number of epochs, are heuristically adjusted, and the models used are shown in Table 4. This process is depicted in Figure 11.

### 6.2. DCNN Result Using STFT

First, to train the DCNN model, we use STFT spectrogram as input and reference depth data as output. The DCNN RMSE results for STFT, encompassing all CPMs and depths for each case, are shown in Table 5. When training DCNNs with STFT techniques, the lowest RMSE is observed in Case 1. STFT shows its highest RMSE in Case 2, followed closely by Case 3. The average RMSE of STFT is 0.505 cm.

**Figure 12:**
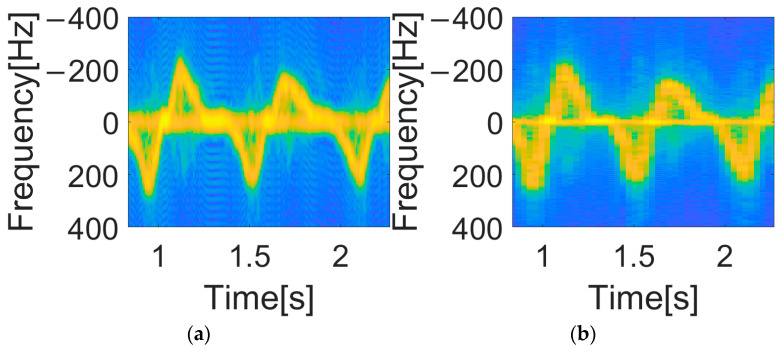
STFT spectrograms with different window lengths: (**a**) window length = 64 (time resolution: 32 ms, frequency resolution: 31.25 Hz), (**b**) window length = 256 (time resolution: 128 ms, frequency resolution: 7.81 Hz).

A major issue with the STFT spectrogram is the window effect. Reducing the size of the window improves the time resolution but, conversely, degrades the frequency resolution. If you increase the size of the window to enhance the Doppler frequency resolution, the time resolution suffers, as seen in Figure 12. Therefore, a high-resolution technique, which is not dominated by window size, is necessary to improve resolution in both time and frequency.

### 6.3. DCNN Result Using WVD

The WVD was originally introduced in the field of quantum mechanics as a bilinear time–frequency representation, and later adopted in signal processing for its ability to provide highly localized joint time–frequency information. Unlike the STFT, which uses fixed windowing and, therefore, suffers from a trade-off between time and frequency resolution, the WVD analyzes the signal’s autocorrelation function across both time and frequency, allowing for the extraction of instantaneous energy with superior resolution. Its nonlinear formulation enables a more accurate depiction of complex and rapidly changing signals [24,30,31]. The corresponding equation is expressed as

(8) $$W\left(t,f\right)=\int_{-\infty }^{\infty }x\left(t+\frac{\tau }{2}\right)\cdot x(t-\frac{\tau }{2})*\cdot e^{-j2\pi f\tau }d\tau$$

However, a well-known limitation of the WVD is the occurrence of cross-term interference when dealing with multi-component signals, which can complicate interpretation in practice [24]. To mitigate this, we employed the smoothed pseudo WVD with a 256-point Hamming window for both time and frequency smoothing.

**Figure 13:**
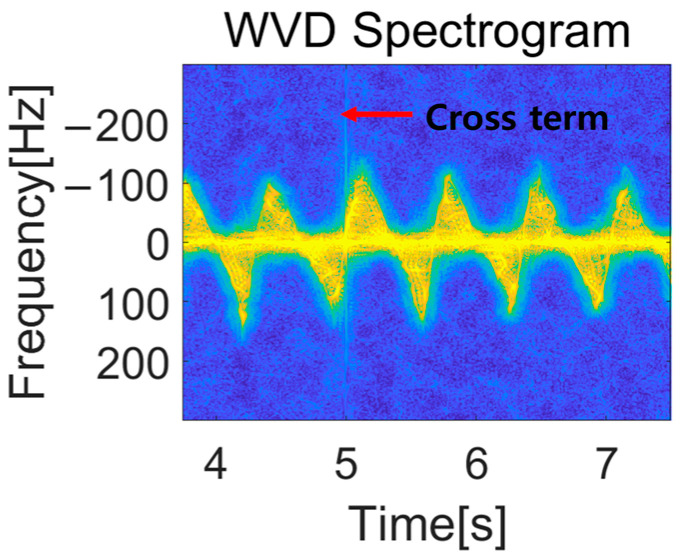
WVD Spectrogram with cross-term.

Figure 13 shows an example of a spectrogram using WVD. It is evident that the spectrogram resolution of WVD is superior to that of STFT. However, in the WVD spectrograms, the influence of cross terms can be seen as unintended lines appearing intermittently throughout the spectrogram. To compare both methods, we trained DCNNs using spectrograms created with both methods. The results are shown in Table 5 and Table 6.

Across the three cases, CNN with WVD outperforms the STFT-based model. Mean RMSE decreases from 0.443 to 0.407 cm in Case 1, from 0.561 to 0.448 cm in Case 2, and from 0.511 to 0.489 cm in Case 3. WVD also shows smaller between-subject variability in Case 2 and Case 3, reflected by lower standard deviations and narrower 95% confidence intervals, while variability in Case 1 is comparable between the two methods. However, a paired *t*-test on the combined dataset indicated that this performance difference lacks sufficient statistical evidence (*t* = 1.4867, *p* = 0.1507).

## 7. Conclusions

In this study, FMCW radar was used to remotely estimate the depth of chest compressions during CPR. The Doppler integration average RMSE for all cases is 0.909 cm. In Cases 2 and 3, the RMSEs were higher, rendering the analysis insignificant. To address this issue, a regression model was utilized to mitigate the depth measurement errors for Cases 2 and 3. Using the regression model, the average RMSE for all cases was 0.535 cm. Subsequently, DCNNs were explored by training with spectrograms from STFT and WVD, and the accuracy of these models was evaluated. The DCNN trained with STFT spectrograms recorded an RMSE of 0.505 cm, while the DCNN trained with WVD spectrograms achieved an RMSE of 0.447 cm, thereby reducing the RMSE by 11% of the AHA-recommended minimum compression depth of 5 cm. This confirmed that the performance of the DCNN based on WVD was significantly superior, indicating that it outperformed the existing regression models as well.

## 8. Discussion

This study shows that FMCW radar combined with deep learning can estimate chest compression depth during CPR without physical contact, which can help support guideline-compliant compressions. The results demonstrate the potential of this approach to support resuscitation in situations where conventional medical equipment is not available. In particular, the use of WVD spectrograms in the DCNN achieved higher accuracy than other approaches, suggesting that high-resolution time–frequency analysis can enhance depth estimation performance.

The best performance achieved an RMSE of about 0.45 cm, which still accounts for more than 40% of the recommended chest compression depth range of 4–5 cm. This indicates that while the method shows promise, the error remains clinically relevant and further improvement is needed. Additionally, a paired *t*-test on the combined dataset indicated that the performance difference between CNN models lacked sufficient statistical evidence (*t* = 1.4867, *p* = 0.1507), highlighting the need for further investigation. So, we will include increasing the sample size and testing under more varied conditions to better evaluate the performance of the WVD-based model.

It is also important to note that our experiments were performed with tripod-mounted radar for stability. In practice, however, smartphone-embedded radar could provide useful measurements without a tripod, as chest compression displacements are several centimeters, larger than body motions of only a few millimeters. Nonetheless, such small motions may still reduce accuracy, especially in handheld use.

Future studies will focus on extending the model to handle various radar angles, applying motion compensation using smartphone sensors, simplifying the model for real-time mobile execution, and synchronizing radar with video for T-CPR integration. We also aim to reduce measurement errors and expand testing across diverse subjects and environments. Validation in realistic emergency scenarios will be required to confirm the practical feasibility of the proposed approach.

## Figures and Tables

**Table 1:** TI AWR1243 FMCW radar parameters.

Configurations	Value
Bandwidth (B)	3990 [MHz]
$\mathrm{Carrier}\;\mathrm{frequency}\;(f_{c})$	77 [GHz]
Number of chirps	3
Number of samples	128
Number of frames	50,000
Period of frame	0.5 [ms]

**Table 2:** Average RMSE (cm) and 95% Confidence Intervals for the Integration Method.

Subject	Radar Position		
	Chest (Case 1)	Side (Case 2)	Head (Case 3)
1	0.474	0.697	0.909
2	0.410	1.250	1.046
3	0.598	0.725	1.353
4	0.519	0.962	1.422
5	0.938	0.686	1.440
6	0.670	0.826	1.309
7	0.446	0.668	1.501
8	0.409	0.653	1.457
Mean	0.558 ± 0.150	0.808 ± 0.172	1.305 ± 0.179
Std.	0.179	0.206	0.214

**Table 3:** Average RMSE (cm) and 95% Confidence Intervals for the Regression Model.

Subject	Radar Position		
	Chest (Case 1)	Side (Case 2)	Head (Case 3)
1	0.479	0.318	0.647
2	0.552	0.452	0.392
3	0.567	0.799	0.723
4	0.365	0.572	0.752
5	0.412	0.727	0.241
6	0.510	0.649	0.364
7	0.753	0.389	0.612
8	0.412	0.643	0.530
Mean	0.506 ± 0.103	0.569 ± 0.141	0.533 ± 0.154
Std.	0.123	0.169	0.184

**Table 4:** DCNN model structure.

1. Image Input Layer	10. A 2D Convolution Layer filter size 3 × 3, 32 filters
2. A 2D Convolution Layer filter size 3 × 3, 8 filters	11. Batch Normalization Layer
3. Batch Normalization Layer	12. ReLU Layer
4. ReLU Layer	13. A 2D Convolution Layer filter size 3 × 3, 32 filters
5. Average Pooling Layer filter size 2 × 2, stride of 2	14. Batch Normalization Layer
6. A 2D Convolution Layer filter size 3 × 3, 32 filters	15. ReLU Layer
7. Batch Normalization Layer	16. Fully Connected Layer of 32
8. ReLU Layer	17. Fully Connected Layer of 1
9. Average Pooling Layer filter size 2 × 2, stride of 2	18. Regression Layer

**Table 5:** RMSE (cm) and 95% Confidence Intervals for the CNN Model Based on STFT.

Subject	Radar Position		
	Chest (Case 1)	Side (Case 2)	Head (Case 3)
1	0.366	0.654	0.664
2	0.481	0.471	0.565
3	0.260	0.800	0.515
4	0.527	0.443	0.483
5	0.356	0.693	0.277
6	0.411	0.313	0.409
7	0.356	0.424	0.455
8	0.786	0.691	0.720
Mean	0.443 ± 0.135	0.561 ± 0.141	0.511 ± 0.154
Std.	0.161	0.169	0.184

**Table 6:** RMSE (cm) and 95% Confidence Intervals for the CNN Model Based on WVD.

Subject	Radar Position		
	Chest (Case 1)	Side (Case 2)	Head (Case 3)
1	0.458	0.363	0.526
2	0.089	0.455	0.621
3	0.455	0.567	0.371
4	0.330	0.680	0.559
5	0.279	0.426	0.447
6	0.505	0.477	0.271
7	0.578	0.316	0.447
8	0.561	0.300	0.653
Mean	0.407 ± 0.138	0.448 ± 0.108	0.489 ± 0.107
Std.	0.165	0.129	0.128

## Data Availability

The dataset analyzed in this study was obtained from proprietary experiments conducted by the authors and is currently not accessible to the public. Interested researchers may request access to the data by contacting the authors directly.
